# Renal function in amyotrophic lateral sclerosis patients on long-term treatment with edaravone

**DOI:** 10.1097/MD.0000000000026127

**Published:** 2021-05-28

**Authors:** Asako Kakimoto, Masatoshi Ishizaki, Hidetsugu Ueyama, Yasushi Maeda, Mitsuharu Ueda

**Affiliations:** a Department of Neurology, National Hospital Organization Kumamoto Saishun Medical Center; b Department of Neurology, Konishi Daiichi Hospital, Fukuoka; c Department of Neurology, Graduate School of Medical Sciences, Kumamoto University, Kumamoto, Japan.

**Keywords:** amyotrophic lateral sclerosis, edaravone, renal function

## Abstract

Edaravone, a free radical-scavenger, was approved in Japan for the treatment of amyotrophic lateral sclerosis (ALS). However, the effect of the drug on renal function in ALS patients remains unclear. This study aimed to investigate renal function in ALS patients on long-term treatment with edaravone by measuring the serum estimated glomerular filtration rate based on cystatin C (eGFR-CysC).

In a retrospective study, the data of ALS patients who were treated with over 10 cycles of intravenous edaravone treatment and were evaluated by eGFR-CysC before and after 10 cycles of treatment between July 2015 and June 2018 were analyzed. Then, the results were compared with those of a control ALS group that had never been treated with edaravone.

There were 11 patients with ALS who received over 10 cycles of intravenous edaravone treatment. The mean interval between the first and final eGFR-CysC measurements was 18.7 ± 7.9 months. Three patients (27.3%) had >20 mL/min/1.73 m^2^ decrease in serum eGFR-CysC. However, no patients discontinued edaravone treatment because of renal dysfunction. The average variation rate of eGFR-CysC was not different between the long-term edaravone group (0.29 ± 1.07) and the control group (–0.34 ± 0.40).

This retrospective, single-center analysis showed no clinical exacerbation of renal function in ALS patients who received long-term treatment with edaravone.

## 1 Introduction

Amyotrophic lateral sclerosis (ALS) is a chronic progressive, fatal motor neuron disease that causes decrease of strength in the upper and lower extremities, bulbar palsy, and respiratory muscles.^[1,2]^ Recent studies showed increased concentrations of 3-nytrothirosin (3-NT), a specific marker of oxidative stress, in ALS patients.^[3]^ Ikawa et al^[4]^ reported that the magnitude of oxidative stress correlated with clinical severity, indicating that it may be related to neurodegenerative changes in ALS.

Edaravone (3-methyl-1-phenyl-2-pyrazolin-5-one; Mitsubishi Tanabe Pharma Corporation, Tokyo, Japan), a free radical scavenger, was shown to be associated with a suppressing motor decline and a concurrent cerebrospinal fluid reduction of 3-NT.^[5]^ In the first placebo-controlled phase 3 study, edaravone showed no significant difference in the ALS functional ration scale-revised (ALSFRS-R) score compared with placebo.^[6]^ However, on post hoc analyses, early-stage ALS patients showed a significantly smaller decrease of the ALSFRS-R score compared with placebo.^[7]^ From these findings, edaravone was approved in Japan for the treatment of ALS in 2015.^[8]^ A recent meta-analysis that analyzed 3 randomized, controlled trials showed that intravenous edaravone was efficacious against ALS patients with no serious side effects.^[9]^ We previously reported a single-center analysis, which suggested slower progression and better prognosis of ALS patients with long-term edaravone treatment in Japan.^[10]^

Meanwhile, the major side effects of this drug are renal dysfunction, hepatic dysfunction, rhabdomyolysis, and acute lung injury.^[11]^ In particular, renal dysfunction can become fatal and is a serious problem in continuing medication for long-term treatment.^[12]^ No reports are available on renal function in ALS patients who received long-term treatment with edaravone. Serum creatinine is generally used as a marker of renal function. However, this marker is inappropriate for ALS patients because they have decreased muscle mass. A previous study showed the potential usefulness of the serum estimated glomerular filtration rate based on cystatin C (eGFR-CysC), which was sensitive and unaffected by muscle volume biomarkers of renal function in ALS patients.^[13]^

This study aimed to investigate renal function in ALS patients on long-term treatment with edaravone by measuring serum eGFR-CysC levels.

## 2 Material and methods

### 2.1 Protocol approvals

This study was approved by the institutional ethics committee at Kumamoto Saishun Medical Center. Oral, informed consent was obtained from each patient before enrolment and participation in the study.

### 2.2 Patients and study design

This was a retrospective study of ALS patients who were treated with over 10 cycles of edaravone treatment and were evaluated by eGFR-CysC before and after 10 cycles of treatment between July 2015 and June 2018 at Kumamoto Saishun Medical Center in Japan. All patients were diagnosed with ALS according to the revised EI Escorial criteria.^[14]^ Patients were administered edaravone (60 mg/d) for 14 consecutive days (first cycle), in conjunction with its discontinuation for 2 weeks. Subsequently, they were administered edaravone for 10 days within a 2-week period followed by a 2-week drug-free period (subsequent cycles) according to the standard protocol.

Clinical features (sex, age, disease duration, ALS severity, eGFR-CysC, use of percutaneous endoscopic gastrostomy, use of ventilator) and the details of edaravone treatment (cycles of administration, place of treatment, reasons for discontinuation in the target period) of 11 patients with ALS who were treated with edaravone at the start of treatment were obtained from patients’ medical records. Then, renal function was compared between the long-term treatment and the control groups (n = 15). The control group included ALS patients at the center who were untreated with edaravone and whose eGFR-CysC was measured at more than a year interval between 2009 and 2017.

### 2.3 Statistics

Statistical analysis was conducted using JMP 9.0 (SAS Institute, Inc., Cary, NC). Statistical comparisons were performed using 2-factor analysis of variance followed by Wilcoxon analysis. Significance was established at *P* < .05.

## 3 Results

**Figure 1:**
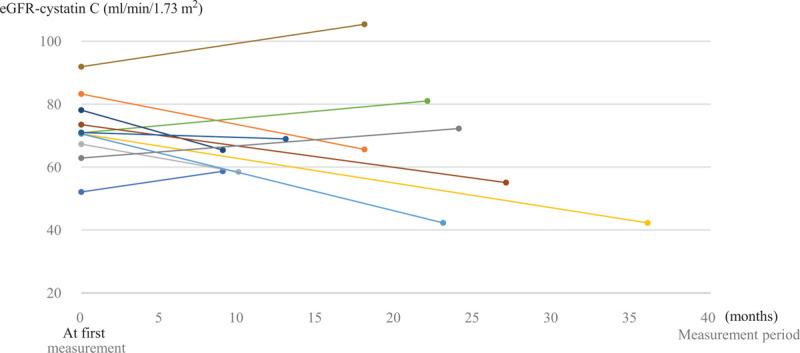
Changes in renal function as reflected by serum eGFR-CysC levels in ALS patients who received long-term edaravone treatment. ALS = amyotrophic lateral sclerosis, eGFR-CysC = the serum estimated glomerular filtration rate based on cystatin C.

Eleven patients (4 men, 7 women) with ALS received over 10 cycles of treatment with intravenous edaravone between July 2015 and June 2018. Their mean age was 65.5 ± 7.8 years (range 39–72 years). Their disease duration was 33.2 ± 21.3 months at the time edaravone was started. Their grades in the Japan ALS severity classification system were from 2 to 5. Patients with grade 5 treated with gastrostomy feeding or tracheostomy and positive pressure ventilation, and non-invasive positive pressure ventilation were the most common, at 45.5%. The mean number of cycles of edaravone administered was 20.7 ± 8.7 (range 11–37). Patients continued treatment with edaravone at a hospital (63.7%) and home-visit care (36.4%). Three of 11 patients stopped treatment with edaravone in this period due to difficulty with vascular access. There were no cases of death or discontinuation of edaravone treatment due to renal dysfunction (Table 1). Changes in renal function were investigated in ALS patients given long-term edaravone treatment (≥10 cycles) using the serum eGFR-CysC levels at the first and final measurements in the period. The mean period between the first and final measurements was 19.0 ± 8.1 months. Eight patients (72.7%) had <20 mL/min/1.73 m^2^ decrease in serum eGFR-CysC. Three patients (27.3%) had >20 mL/min/1.73 m^2^ decrease in serum eGFR-CysC (Fig. 1).

**Table 1:** Clinical characteristics of ALS patients treated with over 10 cycles of edaravone.

Age, y	65.5 ± 7.8
≥65 years	6 (54.5%)
Disease duration, mo	33.2 ± 21.3
ALS severity	
Grade 1	0 (0%)
Grade 2	5 (45.5%)
Grade 3	0 (0%)
Grade 4	1 (9.1%)
Grade 5	5 (45.5%)
Use of PEG	4 (36.4%)
Use of NPPV	4 (36.4%)
Use of TPPV	0 (0%)
Number of cycles of edaravone	20.7 ± 8.7
Place of edaravone treatment	
Hospital	7 (63.7%)
Home	4 (36.4%)
Number who discontinued edaravone treatment	3 (27.3%)
Reasons for discontinuation	
Difficulty in vascular access	3 (100%)

**Figure 2:**
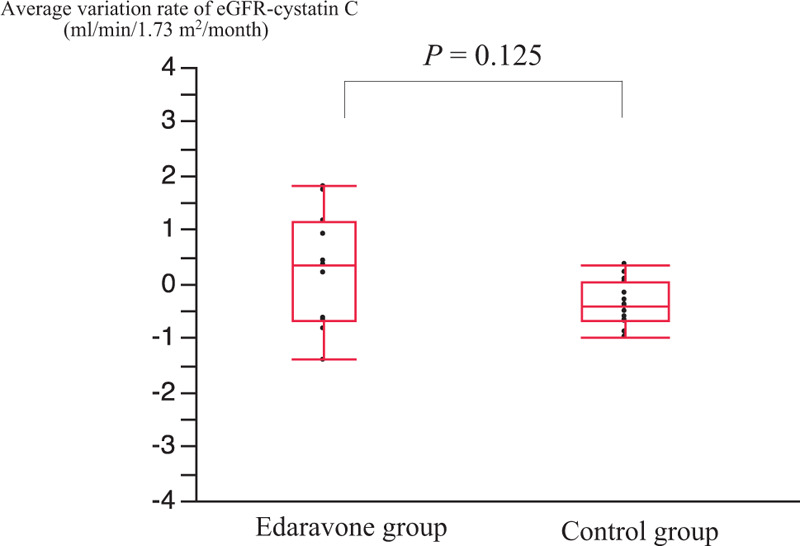
Average variation rate of eGFR-CysC in the edaravone group and the control ALS group. ALS = amyotrophic lateral sclerosis, eGFR-CysC = the serum estimated glomerular filtration rate based on cystatin C, n.s. = not significant.

The renal function of ALS patients long-term treated with edaravone and the control group were also compared. Fifteen consecutive ALS patients (7 men, 8 women; mean age, 61.6 ± 10.5 years) were the control group that was not treated with edaravone. Serum eGFR-CysC was evaluated for at least a year. The edaravone group and the control group had no significant differences in mean age and sex distribution. Patients with grade 5 of the Japan ALS severity classification were significantly more common in the control group (*P* < .01). The first and final serum eGFR-CysC levels were higher in the long-term treatment group (first 73.2 ± 10.9 mL/min/1.73 m^2^, final 68.5 ± 16.6 mL/min/1.73 m^2^) than in the control group (first 57.4 ± 9.9 mL/min/1.73 m^2^; final 49.3 ± 13.2 mL/min/1.73 m^2^; *P* < .01). However, the average time between the 2 measurement points was not different between the long-treatment group (18.7 ± 7.9 months) and the control group (24.4 ± 7.3 months). The average variation rate of eGFR-CysC was not different between the long-treatment group (0.29 ± 1.07) and the control group (–0.34 ± 0.40) (Table 2, Fig. 2).

**Table 2:** A comparison of renal function between the edaravone group and the control ALS group.

	Edaravone group (n = 11)	Control group (n = 15)	*P* value
Sex (male)	4 (36.4%)	7 (46.7%)	.599
Age, y	65.5 ± 7.8	61.6 ± 10.5	.405
Disease duration, y	33.2 ± 21.3	66.7 ± 36.8	<.05
ALS severity grade 5	5 (45.5%)	14 (93.3%)	<.01
Time between 2 measurement points, mo	18.7 ± 7.9	24.4 ± 0.2	.064
eGFR-cystatin C (mL/min/1.73 m^2^) (first measurement)	73.2 ± 10.9	57.4 ± 9.9	<.01
eGFR-cystatin C (mL/min/1.73 m^2^) (final measurement)	68.5 ± 16.6	49.3 ± 13.2	<.01
Average variation rate of eGFR-cystatin C (mL/min/1.73 m^2^/mo)	0.29 ± 1.07	–0.34 ± 0.40	125

## 4 Discussion

This retrospective, single-center analysis showed no significant difference in the average variation rate of eGFR-CysC between long-term edaravone treatment and control ALS groups.

In recent studies, the efficacy and safety of edaravone in the treatment of ALS were shown.^[9,15]^ The open-label study in Korea reported that 16 ALS patients treated with 6 cycles experienced no significant adverse events.^[15]^ A meta-analysis of 183 patients treated by edaravone for 24 weeks showed an efficacious result without any significant adverse events. Furthermore, there was no significant difference between the groups in the number of serious side effects.^[9]^ However, to the best of our knowledge, the effect on renal function of long-term treatment with edaravone in ALS patients has not been reported. Furthermore, serum creatinine as a marker of renal function is not accurate in patients with neuromuscular disease due to the reduced muscle mass.^[16]^ In this paper, the serum eGFR-CysC values of 11 ALS patients who received long-term treatment (mean number of cycles of administration 20.7 ± 8.7) with edaravone were evaluated. A decrease in eGFR-CysC was found in 4 of 11 ALS patients, but the 4 patients did not develop acute renal failure or any clinical manifestations of renal dysfunction (oliguria, edema, hyperkalemia, metabolic acidosis). They were grade 5 in the Japan ALS severity classification. Therefore, the long-term treatment group was compared with a control group including ALS patients not treated with edaravone, and eGFR-CysC was measured at >1 year. The renal function of both groups decreased mildly at 1 or 2 years. As the reasons, repetitive infections, side effects of antibiotics, dehydration, and aging may have affected renal function.

This study has some limitations. First, the clinical features of the long-term treatment group were different from those of the control group. ALS severity was significantly higher in the control group than in the long-term treatment group. However, with more severe disease in the control group, it would be expected that renal function would worsen more, but there was no significant discrepancy between the 2 groups. Second, this study involved a limited number of ALS patients in a single hospital. Thus, a prospective, multicenter study of a large number of ALS patients including a more appropriate control group is needed to confirm any adverse effect of edaravone on renal function.

## 5 Conclusion

In ALS patients who received long-term treatment with edaravone, no clinical exacerbation of renal function was found.

## Author contributions

**Conceptualization:** Hidetsugu Ueyama.

**Data curation:** Hidetsugu Ueyama, Yasushi Maeda.

**Formal analysis:** Yasushi Maeda.

**Supervision:** Mitsuharu Ueda.

**Writing – original draft:** Asako Kakimoto, Masatoshi Ishizaki.

**Writing – review & editing:** Masatoshi Ishizaki, Hidetsugu Ueyama.

## References

[1] Hardiman O Al-Chalabi A Chio A. Correction: amyotrophic lateral sclerosis. Nat Rev Dis Primers 2017;3:17085. 29052611 10.1038/nrdp.2017.85

[2] Renton AE Chiò A Traynor BJ. State of play in amyotrophic lateral sclerosis genetics. Nat Neurosci 2014;17:17–23. 24369373 10.1038/nn.3584 PMC4544832

[3] Beal MF Ferrante RJ Browne SE Matthews RT Kowall NW Brown RH. Increased 3-nitrotyrosine in both sporadic and familial amyotrophic lateral sclerosis. Ann Neurol 1997;42:644–54. 9382477 10.1002/ana.410420416

[4] Ikawa M Okazawa H Tsujikawa T. Increased oxidative stress is related to disease severity in the ALS motor cortex: a PET Study. Neurology 2015;84:2033–9. 25904686 10.1212/WNL.0000000000001588

[5] Yoshino H Kimura A. Investigation of the therapeutic effects of edaravone, a free radical scavenger, on amyotrophic lateral sclerosis (Phase II study). Amyotroph Lateral Scler 2009;7:247–51. 10.1080/17482960600881870 17127563

[6] Abe K Itoyama Y Sobue G. Confirmatory double-blind, parallel-group, placebo-controlled study of efficacy and safety of edaravone (MCI-186) in amyotrophic lateral sclerosis patients. Amyotroph Lateral Scler Frontotemporal Degener 2014;15:610–7. 25286015 10.3109/21678421.2014.959024 PMC4266079

[7] Writing G Edaravone ALSSG. Safety and efficacy of edaravone in well defined patients with amyotrophic lateral sclerosis: a randomised, double-blind, placebo-controlled trial. Lancet Neurol 2017;16:505–12. 28522181 10.1016/S1474-4422(17)30115-1

[8] Corporation MTP, Mitsubishi Tanabe Pharma Corporation Receives Approval for Additional Indication for ALS in Japan; 2015:6205.

[9] Luo L Song Z Li X. Efficacy and safety of edaravone in treatment of amyotrophic lateral sclerosis-a systematic review and meta-analysis. Neurol Sci 2019;40:235–41. 30483992 10.1007/s10072-018-3653-2

[10] Okada M Yamashita S Ueyama H Ishizaki M Maeda Y Ando Y. Long-term effects of edaravone on survival of patients with amyotrophic lateral sclerosis. eNeurologicalSci 2018;11:11–4. 29928711 10.1016/j.ensci.2018.05.001 PMC6006910

[11] RADICUT Injection 30 mg Japanese package insert June 2015 (18th version).

[12] Hishida A. Clinical analysis of 207 patients who developed renal disorders during or after treatment with edaravone reported during post-marketing surveillance. Clin Exp Nephrol 2007;11:292–6. 18085390 10.1007/s10157-007-0495-2

[13] Tetsuka S Morita M Ikeguchi K Nakano I. Utility of cystatin C for renal function in amyotrophic lateral sclerosis. Acta Neurol Scand 2013;128:386–90. 23802939 10.1111/ane.12134

[14] Brooks BR Miller RG Swash M Munsat TL. World Federation of Neurology Research Group on Motor Neuron Diseases. El Escorial revisited: revised criteria for the diagnosis of amyotrophic lateral sclerosis. Amyotroph Lateral Scler Other Motor Neuron Disord 2005;1:293–9. 10.1080/146608200300079536 11464847

[15] Park J-M Kim S-Y Park D Park JS. Effect of edaravone therapy in Korean amyotrophic lateral sclerosis (ALS) patients. Neurol Sci 2020;41:119–23. 31471712 10.1007/s10072-019-04055-3 PMC7223963

[16] Viollet L Gailey S Thornton DJ. Utility of cystatin C to monitor renal function in Duchenne muscular dystrophy. Muscle Nerve 2009;40:438–42. 19623638 10.1002/mus.21420 PMC2740988

